# Early Prediction of MMSE & CSI‐D Scores with AI

**DOI:** 10.1002/alz70861_108631

**Published:** 2025-12-23

**Authors:** Daniel Crovo, Paola Ruiz Puentes, Néstor F. González García, Pablo Arbeláez, Ram Mukunda

**Affiliations:** ^1^ IGC Pharma, Bogotá, Bogotá D.C Colombia; ^2^ SYNDEO INTERNATIONAL S.A.S, Bogotá, Bogotá D.C Colombia; ^3^ IGC Pharma, Potomac, MD USA

## Abstract

**Background:**

We use Llama a LLM to predict MMSE and CSI‐D scores using socio economic data. The growing global burden of dementia needs improved early detection methods that account for diverse socioeconomic factors. While traditional machine learning approaches have been applied to predict cognitive status, they face limitations with complex, heterogeneous data and missing values. Large Language Models (LLMs) are the best performing models in multiple domains, we use Llama a LLM to predict cognitive decline using socioeconomic data.

**Method:**

We developed a data transformation pipeline converting structured tabular data from the Mexican Health and Aging Study (MHAS) into text representations describing each patient data across waves for LLM processing. Our approach used the complete MHAS dataset (26,839 participants across six waves, 2001‐2021) for pretraining, followed by fine‐tuning on the Mexican Cognitive Aging Ancillary Study (Mex‐Cog) subsample (*n* = 3,672) to predict cognitive assessment scores. We compared Random Forest regression (baseline), BERT models with various pretraining configurations, and LLaMA 3.2 1B with multiple adaptation strategies, evaluating performance using Root Mean Squared Error (RMSE).

**Result:**

LLaMA models demonstrated superior performance compared to other LLMs, particularly when pretrained on MHAS data. The LLaMA model with MHAS pretraining achieved the best performance (RMSE 3.14 for MMSE), matching the Random Forest baseline. LLaMA models also excelled in predicting CSI‐D scales, with the MHAS‐pretrained version achieving the lowest RMSE across all models for CSI‐D 6 (0.59) and CSI‐D Informant (4.76). Even without pretraining, LLaMA outperformed BERT variants. The improved performance with pretraining demonstrates that domain‐specific knowledge acquisition significantly enhances LLaMA's predictive capabilities for cognitive assessment.

**Conclusion:**

LLaMA was effective in predicting MMSE scores using socioeconomic data alone (RMSE=3.14) and was also effective in predicting CSID scores (RMSE=0.59). The model can be powerful in helping doctors in resource constrained areas to help predict cognitive decline based on risk factors. Future work will focus on enhancing model interpretability to identify which socioeconomic factors most influence predictions, incorporating multiple databases and multimodal inputs and testing larger model variants to fully leverage the scaling benefits of LLMs for cognitive decline prediction across diverse populations.

